# Dermoscopy use in UK primary care: a survey of GPs with a special interest in dermatology

**DOI:** 10.1111/jdv.15614

**Published:** 2019-05-17

**Authors:** O.T. Jones, L.C. Jurascheck, M. Utukuri, M.M. Pannebakker, J. Emery, F.M. Walter

**Affiliations:** ^1^ The Primary Care Unit Department of Public Health and Primary Care University of Cambridge Cambridge UK; ^2^ Clinical School University of Cambridge Cambridge UK; ^3^ Faculty of Medicine Dentistry and Health Science Department of General Practice and the Centre for Cancer Research Victorian Comprehensive Cancer Centre University of Melbourne Melbourne Australia

## Abstract

**Background:**

Melanoma accounts for 90% of skin cancer mortality and typically presents in primary care, where it can be challenging to distinguish from benign lesions. Dermoscopy is a tool for skin visualization that is routinely used for melanoma diagnosis in secondary care. However, the role of dermoscopy in primary care remains unclear.

**Objectives:**

To determine views on, and use of, dermoscopy by dermatology‐interested general practitioners (GPs).

**Methods:**

An online questionnaire was emailed to the UK Primary Care Dermatology Society members in February 2018, and responses collected over the following 4 weeks.

**Results:**

A total of 205 responses were analysed. Most respondents were GPs (94%), aged over 50 (53%), had a postgraduate dermatological qualification (67%) and used dermoscopy regularly when reviewing pigmented skin lesions (97%). Dermoscopy use was commoner amongst GPs who had worked longer in primary care and had experience of secondary care dermatology. Most had undertaken training in dermoscopy (91%), although one‐fifth (20%) had not updated their training in over 5 years. Most of those who had received only 1 day of face‐to‐face training reported feeling confident using a dermatoscope. Few respondents (11%) reported access to teledermatology or teledermoscopy for urgent or routine referrals.

**Conclusions:**

UK GPs with a special interest in dermatology are routinely using dermoscopy in the primary care setting. More research is needed to establish optimal approaches to training and updating GP dermoscopy skills. When dermoscopy has been shown to be safe, effective, acceptable and cost‐effective in this setting, more GPs may also be able to gain and maintain the skills to implement dermoscopy into routine primary care. Technological advances, including incorporation of artificial intelligence (AI) and algorithms to guide GPs, could also contribute to widening use of dermoscopy among GPs.

## Introduction

Cutaneous malignant melanoma (‘melanoma’) accounts for 90% of all skin cancer mortality.^1^ The incidence has been increasing rapidly in most developed countries for the past several decades,^2^, ^3^ particularly where Caucasian populations are frequently exposed to sunlight^4^; in the UK, incidence has quadrupled since the 1970s.^5^ If melanoma can be detected and surgically treated before metastatic spread occurs, prognosis is greatly improved.^1^ The most likely setting for first presentation and early detection is primary care (>84% in English routinely collected data^6^), and general practitioners (GPs), therefore, need to be able to triage pigmented skin lesions for appropriate referral and management, or safe follow‐up.

Unfortunately, distinguishing between melanoma and benign pigmented skin lesions clinically is challenging. Diagnostic aids for melanoma diagnosis in primary care are clearly desirable, but tools that improve diagnostic accuracy in dermatology settings may not be appropriate for primary care where the prevalence of melanoma is low. A randomized controlled trial set in English primary care found that the use of one such tool, the MoleMate system incorporating SIAscopy with a primary care algorithm, was as accurate as routine care but increased the number of referrals.^7^ Furthermore, even if diagnostic aids do show efficacy in appropriate trial settings, they must be cost‐effective and acceptable to GPs and patients if they are to be integrated effectively into routine practice.

Dermoscopy–also known as dermatoscopy, surface microscopy and epiluminescence microscopy–is a non‐invasive diagnostic aid for evaluation of skin lesions using a hand‐held magnifying lens and immersion fluid, polarization, or both, to eliminate surface reflection at the skin–air interface and closely visualize the skin.^8^ This allows better visualization of sub‐surface structures and colours.^9^ When used by expert dermatologists, dermoscopy shows superior sensitivity to naked eye examination for melanoma detection, without a decrease in specificity.^10^, ^11^, ^12^ In specialist care, its use is, therefore, recommended by UK and other European guidelines,^1^, ^13^ and data from Butler *et al*.^14^ have showed that regular clinical use of dermoscopy amongst UK dermatologists increased from 54.0% in 2003 to 98.5% in 2012. Dermoscopy is not currently recommended for use by GPs in the UK,^15^ although it is used routinely by GPs in Australia,^16^ which has the highest incidence of melanoma worldwide.

The evidence for dermoscopy use by expert dermatologists may not translate to primary care where clinicians have less experience and training in pigmented skin lesion diagnosis and dermoscopy. In this setting, evidence for the diagnostic accuracy of dermoscopy is limited. Our group has recently undertaken a systematic review of the evidence for dermoscopy use in primary care (paper in submission^17^), despite a limited evidence base the results suggest dermoscopy, with training, improves diagnostic accuracy for melanoma and benign lesions and reduced unnecessary excisions and referrals. The extent of training required for a meaningful improvement in performance is unclear, with some studies showing improved diagnostic accuracy after only short training interventions.^18^, ^19^, ^20^, ^21^ It is clear that some training is necessary; even amongst dermatologists, dermoscopy without training performs no better than naked eye examination.^11^, ^22^, ^23^

Previous studies have surveyed the frequency of dermatoscope use and attitudes towards dermoscopy of primary care practitioners (PCPs) in Australia^16^ and France,^24^ finding that 34% and 8%, respectively, use dermoscopy; however, there are no such data from GPs in the UK. We therefore aimed to perform a national survey of UK GP views on dermoscopy and chose to set this among dermatology‐interested GPs, as they are most likely to have implemented dermoscopy use in primary care.

## Methods

### Participants

An online questionnaire was emailed via the Primary Care Dermatology Society to their regular mailing list, comprising approximately 1600 UK GPs and other PCPs (see Table 1). The Primary Care Dermatology Society membership consists mainly of GPs, but is open to any primary care practitioner, is affiliated to the British Association of Dermatologists and delivers educational events and online learning. The questionnaire was available for respondents between 13 February and 13 March 2018. The survey took approximately five minutes to fully complete, and responses were voluntary and anonymous. Respondents could optionally enter a prize draw for online shopping gift cards, but were not otherwise compensated.

**Table 1:** Characteristics of respondents and their practices (n = 205 except where otherwise stated)

Respondent			
Gender (n = 204, missing = 1)	Female		113 (55.4%)
	Male		91 (44.6%)
Age	<40		33 (16.1%)
	41–50		63 (30.7%)
	51–60		80 (39.0%)
	>60		29 (14.1%)
Clinical role	Current GP		192 (93.7%)
	GP trainee		5 (2.4%)
	Retired GP		3 (1.5%)
	Other*		5 (2.4%)
Hospital dermatology post (n = 204, missing = 1)	No		76 (37.3%)
	Yes		
	>6 months		30 (14.7%)
	<6 months		27 (13.2%)
	GP with Extended Role		71 (34.8%)
Dermatology qualifications (multiple responses possible)	No		67 (32.7%)
	Yes		138 (67.3%)
	MSc (1 year, full time)		3
	Postgraduate Diploma (1 year, part time)		32
	Postgraduate Certificate (4 months, part time)		102
	Other (not specified)		6
Confidence managing pigmented skin lesions	Very confident		42 (20.5%)
	Confident		140 (68.3%)
	Neither		20 (9.8%)
	Unconfident		3 (1.5%)
**Their practice**			
Software system	SystmOne		70 (34.1%)
	EMIS/EMIS Web		109 (53.2%)
	Vision		24 (11.7%)
	Other		2 (1.0%)
Region	England	Midlands & East	38 (18.5%)
		North	59 (28.8%)
		South	57 (27.8%)
		London	21 (10.2%)
	Northern Ireland		4 (2.0%)
	Scotland		21 (10.2%)
	Wales		5 (2.4%)

* 2 aesthetic doctors, 1 associate specialist, 1 community specialist, 1 clinical assistant.

### Survey instrument

The questionnaire (reproduced in full in Appendix S1) was adapted from a previous survey^25^ assessing attitudes of primary care physicians towards cancer care and also included new questions generated specifically for this study and informed by expert opinion. A previous pilot of this survey (*n* = 77) addressing the use of checklists and dermoscopy for pigmented skin lesion diagnosis in primary care was emailed to the Royal College of General Practitioner's weekly mailing list. This pilot survey was subsequently refined to focus on dermoscopy use.

In this final version, there were 26 questions in three sections. The first section (9 questions) focused on demographics, using questions from the previous surveys regarding age and gender, new items were added to characterize experience with dermatology and skin cancer. The second section (4 questions) focused on the characteristics of the respondent's general practice, with items from the previous surveys regarding practice location, primary care software and use of checklists for 2‐week wait referral pathways for suspicious pigmented skin lesions. The third section (13 questions) focused on use of dermoscopy and teledermoscopy; it included questions from the previous surveys regarding dermoscopy use, use of dermoscopy checklists, confidence with dermoscopy use, extent and modality of previous dermoscopy training, dermoscopy use within the practice; and also newly developed questions on the availability of teledermatology and teledermoscopy for urgent and routine dermatology referrals.

Quantitative survey items utilized ‘yes/no’ responses and five‐point Likert scales for the frequency of dermoscopy use (1 = never to 5 = every time) and the self‐reported confidence in pigmented skin lesion diagnosis and dermoscopy use (1 = very confident to 5 = very unconfident). Some survey items allowed free text responses regarding type and duration of training.

### Statistical analysis

Responses were collected via Qualtrics (2018) and exported to Microsoft Excel (2016). Free text responses were manually parsed and, where possible, grouped into discrete categories. Geographical locations of practices were categorized into NHS trust and health boards according to official NHS structural information for each of the countries of the UK.^26^, ^27^, ^28^, ^29^ English NHS trusts were then categorized into North, South, Midlands and East, and London mirroring allocation in the NHS digital statistical analysis of general and personal medical services.^30^ For the various training modalities reported, the following categories were used: face‐to‐face training, online course training; self‐directed study; hands‐on learning; and short didactic training. Statistical analysis was mainly descriptive. Cross‐tab analysis was undertaken to investigate the relationships between certain variables, these included years worked in primary care vs use of dermoscopy, length of hospital dermatology post vs use of dermoscopy and length of face‐to‐face training course vs confidence.

## Results

### Sample characteristics

A total of 214 responded to the survey, but 9 were less than 50% complete and were excluded, giving a final sample of 205 respondents. If all 1600 people on the PCDS mailing list received and read the email, this suggests a response rate of 13%; however, it is likely that some emails did not reach or were not read by members, therefore this response rate is likely to be an underestimate.

Table 1 shows the characteristics of respondents and their practices. Most respondents were older and more experienced than the national average, with 53.1% of our respondents aged 51 and over. In comparison, 33.6% of English GPs are 50 or older.^30^ The vast majority of respondents were GPs (93.7%).

Most respondents had previous experience in dermatology; 62.7% had previously held either a hospital dermatology or GP with extended role (GPwER) post (previously GP with a special interest, GPwSI) and 67.3% had at least one dermatology‐related postgraduate qualification. About 88.8% reported feeling confident in managing patients presenting with pigmented skin lesions. Although in this select population of dermatology‐interested GPs, these findings are not necessarily surprising.

About 53.2% reported using EMIS (a leading UK primary care system software provider), in line with other reports that EMIS has 56% of the market share in England for primary care system software.^31^ Geographical location of respondents in England was broadly representative of the GP population in England^30^ with slight under‐representation of NHS London and NHS Midlands and East areas. National representation, compared to BMA data,^32^ showed that Scotland and Northern Ireland were slightly over‐represented in our sample.

### Access to and use of dermoscopy, and confidence in its use

Table 2 shows reported access to, and use of dermoscopy, and confidence in its use. Of those with access to a dermatoscope (>92%), nearly all used it regularly to assess pigmented skin lesions (96.8%) and the majority felt confident in using it (82.1%). About 39.3% had a local colleague using dermoscopy; however, few had referred to them. Five of the six respondents who had access to a dermatoscope but did not use dermoscopy regularly had never held a dermatology or GPwER post and had no postgraduate dermatology qualifications.

**Table 2:** Reported access to, and use of dermoscopy, and confidence in its use (n = 205 except where otherwise stated)

**Ownership of a dermatoscope**	**Yes**		178 (86.8%)
	**No**		27 (13.2%)
**Dermatoscope available at work**	**Yes**		190 (92.7%)
	**No**		15 (7.3%)
**Use of dermoscopy when reviewing pigmented skin lesions (*n* = 190)**	**Yes,**	Every time	164 (86.3%)
		Most of the time	20 (10.5%)
		Sometimes	0 (0%)
		But rarely	4 (2.1%)
	**No, never**		2 (1.1%)
**Confidence in using dermoscopy (*n* = 190)**	**Very confident**		34 (17.9%)
	**Confident**		122 (64.2%)
	**Neither**		26 (13.7%)
	**Unconfident**		8 (4.2%)
**Colleague in practice or locally who uses dermoscopy (*n* = 201, missing = 4)**	**Yes,**	And I refer to them	34 (16.9%)
		But I have never referred	45 (22.4%)
	**Not sure**		9 (4.5%)
	**No**		113 (56.2%)

### Training in dermoscopy use

Table 3 shows training in dermoscopy use. 91.0% of respondents reported receiving dermoscopy training, the modality and length of training was variable, and for 20.3% of these it had been over 5 years since their last dermoscopy training. Face‐to‐face training courses, such as those run by the Primary Care Dermatology Society (PCDS), were by far the most popular training modality (66.5%). The majority of respondents who had attended face‐to‐face training courses had attended for up to a single day (56.5%) or between a day and a week (40.9%). The most popular modality for training updates was self‐directed learning (33.5%), but 36.0% reported that they had never updated their training.

**Table 3:** Training in dermoscopy use

**Training to use dermoscopy (*n* = 201, missing = 4)**	Yes		183 (91.0%)
	No		18 (9.0%)
**Time since last dermoscopy training (*n* = 172, missing = 11)**	<1 year		55 (32.0%)
	1–5 years		82 (47.7%)
	>5 years		35 (20.3%)
**Modality of dermoscopy training (multiple responses possible) (*n* = 182, missing = 1)**	Any face‐to‐face‐course		121
		PCDS Beginners (1 day)	43
		PCDS Advanced (1 day)	16
		Other (not specified)	73
	Online course training (12 weeks)		27
	Hands‐on training		21
	Short didactic training		17
	Self‐directed study		45
**Updates to dermoscopy training (multiple responses possible) (*n* = 161, missing = 22)**	Any face‐to‐face‐course		49
		PCDS Advanced (1 day)	31
		Other (not specified)	24
	Online course training (12 weeks)		0
	Short didactic training		23
	Hands‐on training		11
	Self‐directed study		54
	Not updated skills		58

We calculated the relationship between training and confidence in using dermoscopy and found that 74.6% of those that had received only a single day of face‐to‐face dermoscopy training was confident in its use.

### Use of teledermoscopy and other teledermatology technologies

Table 4 shows the availability of teledermatology (using clinical images) or teledermoscopy (using dermoscopic images) technologies for referral of skin lesions to secondary care. Most respondents reported that for urgent (2‐week wait) referrals, teledermatology (75.5%) or teledermoscopy (79.6%) referrals were not possible locally. 71.2% were unable to use teledermatology or teledermoscopy technologies to refer skin lesions routinely, although there were a few respondents who indicated that these technologies were being piloted or planned soon.

**Table 4:** Use of teledermatology and teledermatology‐related technologies

Does your 2‐week wait referral pathway for suspicious pigmented skin lesions use teledermatology? (*n* = 196, missing = 9)	**Yes**	via digital camera images	17 (8.7%)
		via smartphone images	15 (7.7%)
		via digital video images	1 (0.5%)
		via live video	0 (0%)
	**No**	Not at all	138 (70.4%)
		Previously available but no longer	6 (3.1%)
		Available for other lesions	1 (0.5%)
		But service planned to start soon	3 (1.5%)
	**Not sure**		15 (7.7%)
Does your 2‐week wait referral pathway for suspicious pigmented skin lesions use teledermoscopy? (*n* = 196, missing = 9)	**Yes**	via digital camera images	13 (6.6%)
		via smartphone images	8 (4.1%)
		via digital video images	0 (0%)
		via live video	0 (0%)
	**No**	not at all	153 (78.2%)
		Previously available but no longer	1 (0.5%)
		Unusable due to technical issues	2 (1.0%)
	**Not sure**		19 (9.7%)

## Discussion

### Summary

To our knowledge, this is the first survey investigating the prevalence of dermoscopy use and training amongst UK GPs. Our findings suggest that access to a dermatoscope and its frequent use is commonplace amongst dermatology‐interested UK GPs, who have shown that it is possible to undertake dermoscopy training and successfully incorporate dermoscopy into routine clinical practice. We are now interested whether, with careful and tailored approaches to training and implementation, more GPs might be able to safely implement dermoscopy into routine primary care.

Most respondents were GPs and had a postgraduate dermatological qualification. Nearly all used dermoscopy regularly and with confidence, as might be expected from this special interest group, although 7% reported no access to a dermatoscope. Dermoscopy use was commoner amongst GPs who had worked longer in primary care and had experience of secondary care dermatology. Most respondents had undertaken training in dermoscopy, although one‐fifth had not updated their dermoscopy skills in over 5 years. Clearly, this is an issue for revalidation in these specialist skills. One respondent indicated that having completed a dermoscopy training course, they were ‘under so much pressure at work haven't had time to sit in […] to learn how to use dermatoscope in clinic and gain practical experience’*,* suggesting pressure from clinical responsibilities prevented them from continuing their dermoscopy education. Most of those who had received only one day of face‐to‐face training felt confident using a dermatoscope; however, there is an important distinction between confidence and clinical competence, and research is needed to establish if this level of training produces a measurable improvement in diagnostic accuracy.

Few respondents reported access to teledermoscopy or teledermatology for either urgent (2‐week wait) or routine referrals, suggesting these technologies are not yet widely available. As the recent NHS Long Term Plan^33^ identifies the increased integration of digital technologies as one of the key aims for the NHS, access to teledermatology is clearly one area that could be targeted.

### Strengths and limitations

The current UK policy and clinical drive to encourage GPs to incorporate dermoscopy into their routine practice, based on international but not UK evidence for its effectiveness and cost‐effectiveness, makes this study very topical. Our sample was representative of the UK GP population in terms of gender and geography (including practitioners from all four UK nations), but was older, and enriched by GPs with an interest in dermatology. This was due to our recruitment strategy using the PCDS mailing list. These GPs are the most likely to be early adopters of routine dermoscopy in primary care, and their views on use, access and training needs have relevance for the implementation of dermoscopy across the wider GP community.

The main limitation of this study is that we are uncertain of the denominator, and therefore, we were unable to calculate an accurate response rate or characterise the non‐responders. However, it is likely that GPs with an interest in dermatology and dermoscopy would have been more likely to respond to the survey invitation. While we were able to use validated items for the general questions, we developed new items for the specific questions about dermoscopy use, confidence and training. These were reviewed and revised by the wider study team and agreed to have good face validity, but further psychometric validation may have been desirable. Finally, some practices may have internal referral mechanisms, so that potentially suspicious pigmented skin lesion are reviewed by GPs with dermoscopy or GPwER expertise, this may have affected the responses of GPs in these practices and would not have been fully captured by our survey.

### Comparisons with existing literature

We found almost universal routine dermoscopy use when reviewing pigmented skin lesions (97%). This is likely to be at least partly due to two‐thirds of our respondents reporting a personal history of dermatology posts and qualifications, in keeping with Australian evidence that GP subspecialisation in dermatology leads to increased rates of dermoscopy use.^34^ This very high level of dermoscopy use is closer to that of UK dermatologists in 2012 (99%),^14^ than for primary care practitioners internationally (34% in Australia in 2007^16^ and 8% in France in 2016^24^), or in our pilot survey of UK GPs without a special interest in dermatology (*n* = 77, 17%) (unpublished data). Recent reviews of dermoscopy use for melanoma diagnosis^17^, ^35^ point to an increase in diagnostic accuracy for benign lesions and melanoma when dermoscopy is used by trained GPs, and it can lead to a reduction in unnecessary referrals and excisions. There is also international evidence that GPs are receptive to incorporating dermoscopy into their routine practice.^24^, ^36^, ^37^, ^38^

Diagnostic accuracy for dermoscopy users appears to depend on sufficient training.^11^, ^21^, ^23^ However, there is little evidence to confirm the optimal training modality and duration. Educational interventions in previous randomized controlled trials have ranged from a single hour^21^ to 10 h plus further textbook reading or e‐learning.^39^ However, there is no randomized controlled trial evidence comparing the effects of shorter and longer training periods. Previous studies have found that perceived training requirements are amongst the most commonly cited barriers to implementation of dermoscopy.^17^, ^24^, ^40^ This study suggests that 1 day of face‐to‐face training is sufficient to enable GPs with a special interest in dermatology to feel confident in dermoscopy use. However, further research is needed to evaluate whether this level of training produces a significant improvement in diagnostic accuracy, and whether this approach is also suitable for GPs without a special interest in dermatology. Australian RCT evidence showed high drop‐out rates of GPs from longer dermoscopy training schedules.^41^ Finally, we found that a substantial minority of respondents had not kept their dermoscopy training up to date; this may be of more concern if or when dermoscopy becomes more widely implemented across UK general practice.

### Implications for research and/or practice

This survey shows that dermatology‐interested GPs in the UK are routinely using dermoscopy in the primary care setting, suggesting that it may be feasible for dermoscopy to be more widely implemented in UK primary care. However, further evidence is needed to show that dermoscopy is safe, effective, acceptable and cost‐effective in this setting. The survey results also highlight that further research is needed, particularly around the type and length of dermoscopy training, and refresher courses required, for effective dermoscopy use. Optimal dermoscopy training methods which produce a significant improvement in diagnostic accuracy need to be established, and review is needed on how to best maintain competencies, including how frequently training updates are needed.

Prior to more widespread implementation, providers and policymakers will also need evidence of cost‐effectiveness. Whilst dermoscopy is relatively inexpensive compared with other diagnostic aids such as ultrasound or confocal microscopy, it would still represent a significant cost were it to be recommended for use by every GP in the UK. Establishing the optimal position of dermoscopy in the diagnostic pathway will be important in the cost‐effective assessment, for example whether dermoscopy use in UK primary care should be at an individual level, a practice level or a primary care hub level. One dermoscopy‐trained GP per practice has been advocated by the Primary Care Dermatology Society.^42^ Finally, technological advances, including incorporation of artificial intelligence (AI) for pattern recognition, and algorithms to guide GPs, could also contribute to enhanced use of dermoscopy among GPs.

## Ethics Approval

Not required.

## Supporting information


**Appendix S1.** Questionnaire, reproduced in full.
