# Supplementary material for: Dermoscopy use in UK primary care: a survey of GPs with a special interest in dermatology
Source: J Eur Acad Dermatol Venereol. 2019 May 17;33(9):1706–12. doi: 10.1111/jdv.15614 (PMC6767170; doi:10.1111/jdv.15614)
Supplement: Supplementary file 1 — Appendix S1. Questionnaire, reproduced in full. [file JDV-33-1706-s001.docx]

**Appendix 1** – questionnaire, reproduced in full

**About you**

Q1 How old are you?

- < 31 years
- 31-40 years
- 41-50 years
- 51-60 years
- > 60 years

Q2 Do you work as a:

- GP
- Practice nurse
- Other, please specify

Q3 For how many years have you worked in primary care?

- Current trainee
- 0-10 years
- 11-20 years
- 21-30 years
- > 30 years

Q4 What is your gender?

- Male
- Female
- Prefer not to say

Q5 Have you ever held a Hospital Dermatology Post?

- Yes, for 6 months or less
- Yes, for more than 6 months
- Yes, as a current/past GP with a Special Interest (GPwSI) in dermatology
- No

Q6 Do you have any of the following GP/dermatology qualifications? Please select all that apply.

- Postgraduate dermatology training: Certificate
- Postgraduate dermatology training: Masters
- Other (please specify)
- No dermatological qualifications

Q7 Do you or your GP colleagues excise squamous cell carcinomas (SCCs) in your practice?

- Yes all
- Yes some (please specify)
- No

Q8 Do you or your GP colleagues excise basal cell carcinomas (BCCs) in your practice?

- Yes all
- Yes some (please specify)
- No

Q9 How confident are you in how you manage patients presenting with pigmented skin lesions?

- Very confident
- Confident
- Neither confident nor unconfident
- Unconfident
- Very unconfident

**About your practice**

If you do not regularly practice at one surgery, please think of the practice where you have spent the most time during the last 12 months.

Q10 Which software system do you use in your practice?

- SystmOne
- EMIS/EMIS Web
- Vision
- Other (please specify)

Q11 Which Region is your practice a part of?

Q12 Which NHS trusts do you refer patients to? Please state up to three.

Q13 Does your CCG or NHS Trust use a checklist for the two week wait referral pathway for suspicious pigmented skin lesions?

- Yes (please specify which one if known)
- Not sure
- No

**USE OF DERMOSCOPY**
These questions examine your use of dermoscopy.

Q14 Do you own a dermatoscope?

- Yes
- No

Q15 Is there a dermatoscope available in your practice?

- Yes
- No

Q16 If a dermatoscope is available, do you currently use dermoscopy when reviewing pigmented skin? lesions?

- No, never
- Yes, but rarely
- Yes, sometimes
- Yes, most of the time
- Yes, every time

Q17 If a dermatoscope is available, do you use a checklist to help with your assessment?

- No checklist
- Classic pattern analysis
- CASH
- ABCD for dermoscopy
- 7-point checklist
- The Menzies method
- The 3-point checklist
- The revised pattern analysis
- ‘Chaos and Clues’
- Other

Q18 If 'other' selected, please specify which checklist you use?

Q19 If a dermatoscope is available, do you use a physical or mental checklist?

- No checklist
- Physical checklist
- Mental checklist
- Both physical and mental
- Other (please explain)

Q20 If a dermatoscope is available, how confident are you in using dermoscopy to manage patients presenting with pigmented skin lesions?

- Very confident
- Confident
- Neither confident nor unconfident
- Unconfident
- Very unconfident

Q21 Have you undertaken training to use dermoscopy to review a pigmented skin lesion?

- No
- Yes

Q22 If you have undertaken training to use a dermatoscope, please provide details:

What did you do (e.g. face-to-face course, online learning, practical session etc.)?

How long did the training take?

How long ago was it?

Have you updated your skills (and if yes, how)?

Q23 Do you have a colleague in your practice or locally who uses dermoscopy?

- Yes, and I refer some patients to them
- Yes, but I have never referred a patient to them
- Not sure
- No

Q24 Does you CCG or NHS Trust use tele-dermatology (i.e. use of telecommunication technologies to exchange images of lesions with secondary care) for their two-week wait referral pathway for suspicious pigmented skin lesions?

- Yes, via digital camera images
- Yes, via smartphone images
- Yes, via digital video images
- Yes, via live video link
- Not sure
- No
- Other (please specify)

Q25 Does your CCG or NHS Trust use tele-dermoscopy (i.e. use of a dermatoscope/dermoscopy with telecommunication technologies to exchange images of lesions with secondary care) for their two-week wait referral pathway for suspicious pigmented skin lesions?

- Yes, via digital camera images
- Yes, via smartphone images
- Yes, via digital video images
- Yes, via live video link
- Not sure
- No
- Other (please specify)

Q26 Does your CCG or NHS Trust use tele-dermatology or tele-dermoscopy for their routine referral pathway/s for pigmented skin lesions or other skin lesions?

- Yes, via digital camera images
- Yes, via smartphone images
- Yes, via digital video images
- Yes, via live video link
- Not sure
- No
- Other (please specify)
